# Performance Analysis and Optimization of a Series Heat Exchangers Organic Rankine Cycle Utilizing Multi-Heat Sources from a Marine Diesel Engine

**DOI:** 10.3390/e23070906

**Published:** 2021-07-16

**Authors:** Youyi Li, Tianhao Tang

**Affiliations:** The Institute of Power Drive and Control, Shanghai Maritime University, 1550 Haigang Ave., Shanghai 201306, China; thtang@shmtu.edu.cn

**Keywords:** multi-heat sources, Organic Rankine Cycle, multi-objective optimization, marine diesel engine, thermoeconomic analysis

## Abstract

Organic Rankine Cycle (ORC) is an effective way to recycle waste heat sources of a marine diesel engine. The aim of the present paper is to analyze and optimize the thermoeconomic performance of a Series Heat Exchangers ORC (SHEORC) for recovering energy from jacket water, scavenge air, and exhaust gas. The three sources are combined into three groups of jacket water (JW)→exhaust gas (EG), scavenge air (SA)→exhaust gas, and jacket water→scavenge air→exhaust gas. The influence of fluid mass flow rate, evaporation pressure, and heat source recovery proportion on the thermal performance and economic performance of SHEORC was studied. A single-objective optimization with power output as the objective and multi-objective optimization with exergy efficiency and levelized cost of energy (LCOE) as the objectives are carried out. The analysis results show that in jacket water→exhaust gas and jacket water→scavenge air→exhaust gas source combination, there is an optimal heat recovery proportion through which the SHEORC could obtain the best performance. The optimization results showed that R245ca has the best performance in thermoeconomic performance in all three source combinations. With scavenge air→exhaust, the power output, exergy efficiency, and LCOE are 354.19 kW, 59.02%, and 0.1150 $/kWh, respectively. Integrating the jacket water into the SA→EG group would not increase the power output, but would decrease the LCOE.

## 1. Introduction

Maritime transport occupies a dominant position in world trade, and approximately 80% of world trade is done by merchant ship [1]. The vast majority of seagoing vessels use two-stroke low-speed diesel engines as their main propulsion because the diesel engine can burn the economical heavy fuel oil (HFO). Although diesel engines have very high thermal efficiency, nearly half of the energy in the HFO is still emitted into the environment through exhaust gas, jacket water, scavenging air, lubricating oil, and heat radiation [2]. Utilizing these waste heat sources can cut down the cost of fuel consumption and reduce ${\mathrm{CO}}_{2}$ emissions. Organic Rankine Cycle (ORC) is an effective way to convert a medium-low temperature heat source into electricity [3]. Therefore, using ORC to recover energy from heat sources has received much attention [4]. However, there is more than one heat source on board. Using a single ORC to recover a single heat source will cause higher investment costs [5].

One way to improve the power output and reduce investment cost is utilizing multi-heat sources simultaneously [6]. Vaja and Gambarotta [3] proposed a preheated ORC that could utilize jacket water and exhaust gas together. The jacket water is applied to preheat the working fluid. The results indicated that the preheated ORC has a higher power output than a simple ORC. Ma et al. [7] proposed a cascade utilization method to recover energy from exhaust gas and jacket water simultaneously. The results showed that full recovery of the jacket water with a single ORC loop could have a lower power output. Kim et al. [8] applied a single loop ORC to utilize the jacket water and exhaust gas. Two recuperators are added into the single loop ORC. The modification could change the pinch point position and obtain higher power output. It can be seen from the above studies that utilizing two waste heat sources in one waste heat recovery system (WHRS) could obtain better themoeconomic performance.

Furthermore, to obtain higher power output, three kinds of heat sources from the marine engine are recovered. Yang et al. [9] presented a dual-loop ORC(DLORC) to utilize jacket water, intake air, and exhaust gas. The upper loop of the DLORC is used to recycle the exhaust gas. In the lower cycle, the working fluid is first preheated by the intake air, then heated by the working fluid in the condenser of the upper cycle, and finally heated up into a saturated vapor state by the cooling water. The analysis results showed that the thermal efficiency of the diesel engine integrated with the DLORC increased by 13%. Yang [10] analyzed the thermal and economic performance of a single loop transcritical Rankine cycle to recover heat from the exhaust gas, jacket water and scavenging air. The proposed system shows that utilizing more sources could reduce more ${\mathrm{CO}}_{2}$ emission and the investment cost of the system. Furthermore, Yang [11] utilized heat from the exhaust gas, jacket water, scavenge air, and lubricating oil with the single loop transcritical Rankine cycle. These studies showed that utilizing multi-heat sources could significantly improve the thermal efficiency of the diesel engine.

A review of the literature indicates that only several studies [9,12,13] are presented to recover heat from the multi-heat sources of the marine diesel engine. Moreover, it seems that no study has regarded recovering multi-heat sources with a single loop Organic Rankine cycle to our knowledge. In this research, a Series Heat Exchangers Organic Rankine Cycle (SHEORC) is proposed to recover energy from the exhaust gas, jacket water, and scavenge air. The SHEORC was applied to recycle three multi-heat-source combinations. The three groups are jacket water (JW)→exhaust gas (EG), scavenge air (SA)→exhaust gas, and jacket water→scavenge air→exhaust gas. Thermoeconomic performance analysis and optimization of the SHEORC have been carried out to find the best combination and suitable working fluid.

## 2. Methods

### 2.1. Multi-Heat Sources

The multi-heat sources to be recovered in this study are from a state-of-the-art MAN B&W 6S35ME-B9 diesel engine. This engine is a six-cylinder in-line two-stroke low-speed diesel engine that can be used as the main propulsion of merchant ships sailing on the ocean. The basic parameters of the diesel engine are listed in Table 1.

The mass flow and temperature of the heat sources are measured during field test and are illustrated in Table 2.

The exhaust gas composition is listed in Table 3 when the diesel engine is running at 100% Specified Maximum Continuous Rating (SMCR) and is burning the fuel oil with a sulfur content of 3.5%. The composition parameters are applied to calculate the dew point temperature [14] and properties of the exhaust gas.

### 2.2. Series Heat Exchangers Organic Rankine Cycle

**Figure 1:**
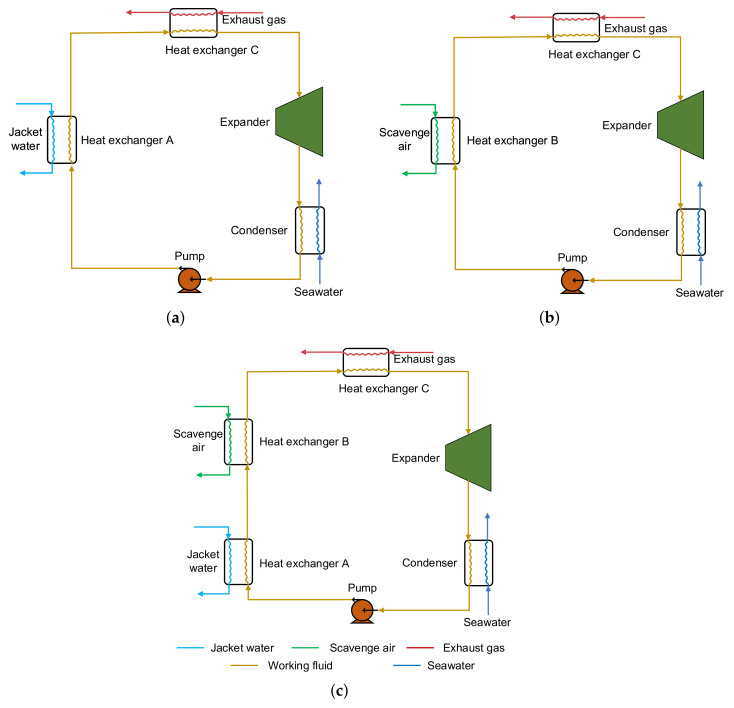
Configurations of the Series Heat Exchangers Organic Rankine Cycle for utilizing multi-heat sources. (**a**) Jacket water→exhaust gas. (**b**) Scavenge air→exhaust gas. (**c**) Jacket water→scavenge air→exhaust gas.

In this research, a Series Heat Exchangers Organic Rankine Cycle (SHEORC) is proposed to harness exhaust gas, scavenge air, and jacket water. Considering that there are three heat resources to be utilized, these three heat sources can be divided into three combinations. The three groups are jacket water→exhaust gas, scavenge air→exhaust gas, and jacket water→scavenge air→exhaust gas. Consequently, the configurations of the SHEORC are shown in Figure 1.

**Figure 2:**
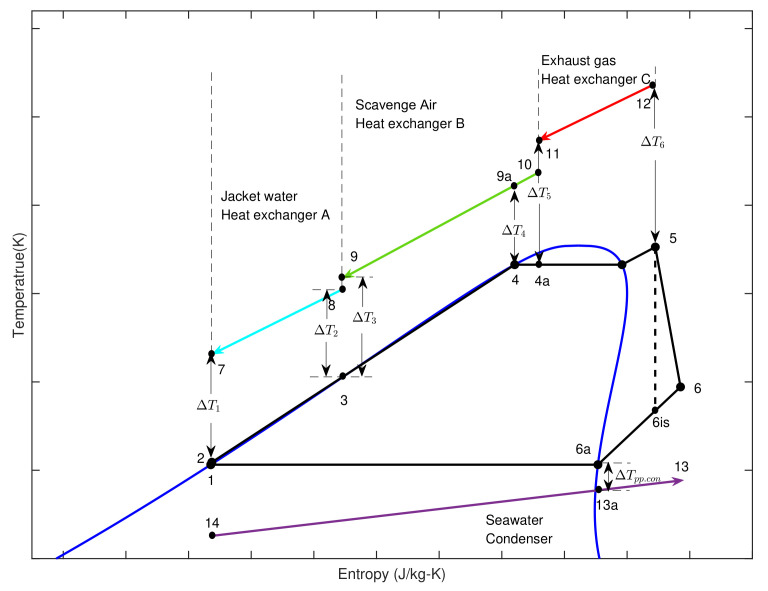
T-s diagram of the SHEORC.

As can be seen in Figure 1, the SHEORC system consists of a working fluid pump, an expander, and a condenser, and several heat exchangers. The number of heat sources determines the number of heat exchangers. For example, in the source combination JW→SA→EG, the working fluid from the pump first flows through the heat exchanger A. The working medium in heat exchanger A is heated by jacket water. Afterward, the working fluid passes through heat exchanger B and recovers the heat from scavenging air. Finally, the working fluid from heat exchanger B is circulated into heat exchanger C. The working fluid absorbs energy from the exhaust gas in the heat exchanger C. The working fluid coming out of the heat exchanger C is high-pressure steam under a superheated state. Then the superheated steam expands in the expander to convert the energy into mechanical energy and turns into low-pressure steam. Subsequently, the steam rejects heat to seawater in the condenser and changes into a saturated liquid. After all, the saturated liquid is then transferred to heat exchanger A by the working fluid pump. The relationships between the temperature and entropy of the integration process are illustrated in Figure 2.

### 2.3. Selection of Working Fluid

In the SHEORC system, working fluids’ thermodynamic and transport properties have considerable influence on thermodynamic and economic performance. Consequently, finding out the optimal working fluid for the SHEORC system is indispensable. To avoid damage to the environment, the candidate working fluids should have zero ozone depletion potential value and low global warming potential. Table 4 presents the properties of the working fluids.

### 2.4. Thermodynamic Modeling

The thermodynamic model of SHEORC includes energy and exergy balances analysis, the detailed model of each component is described as follows.

The energy balance of the heat exchanger A, B, and C can be expressed as follows:

(1) $$\begin{array}{cc} \; & {\dot{Q}}_{\mathrm{eg}}={\dot{m}}_{\mathrm{eg}}c_{p,\mathrm{eg}}\left(T_{\mathrm{eg},\mathrm{in}}-T_{\mathrm{eg},\mathrm{out}}\right) \end{array}$$

(2) $$\begin{array}{cc} \; & {\dot{Q}}_{\mathrm{sa}}={\dot{m}}_{\mathrm{sa}}c_{p,\mathrm{sa}}\left(T_{\mathrm{sa},\mathrm{in}}-T_{\mathrm{sa},\mathrm{out}}\right) \end{array}$$

(3) $$\begin{array}{cc} \; & {\dot{Q}}_{\mathrm{jw}}={\dot{m}}_{\mathrm{jw}}c_{p,\mathrm{jw}}\left(T_{\mathrm{jw},\mathrm{in}}-T_{\mathrm{jw},\mathrm{out}}\right) \end{array}$$

(4) $$\begin{array}{cc} \; & {\dot{Q}}_{\mathrm{HEA}}={\dot{m}}_{r}\left(h_{3}-h_{2}\right) \end{array}$$

(5) $$\begin{array}{cc} \; & {\dot{Q}}_{\mathrm{HEB}}={\dot{m}}_{r}\left(h_{4}-h_{3}\right) \end{array}$$

(6) $$\begin{array}{cc} \; & {\dot{Q}}_{\mathrm{HEC}}={\dot{m}}_{r}\left(h_{5}-h_{4}\right) \end{array}$$

where $\dot{Q}$ is heat transfer rate, $\dot{m}$ is mass flow rate, *T* is temperature, *h* is specific enthalpy of the working fluid, subscript eg is exhaust gas, subscript sa is scavenge air, subscript jw is jacket water, subscript in is inlet, and subscript out is outlet.

The temperature at heat exchanger C outlet is calculated as follows:

(7) $$T_{5}=T_{\mathrm{ev}}+T_{\sup}$$

where $T_{5}$ is temperature of the working fluid at heat exchanger C outlet, $T_{\mathrm{ev}}$ is evaporating temperature, and $T_{\sup}$ is superheat temperature.

The output power of the expander is determined by

(8) $${\dot{W}}_{\exp}={\dot{m}}_{r}\left(h_{5}-h_{6}\right)$$

where ${\dot{W}}_{\exp}$ is the output power of the expander, $h_{5}$ is specific enthalpy of working fluid at the expander inlet, and $h_{6}$ is specific enthalpy of working fluid at the expander outlet.

The energy balance of the condenser can be described by

(9) $$\begin{array}{cc} \; & {\dot{Q}}_{\mathrm{con}}={\dot{m}}_{r}\left(h_{6}-h_{1}\right) \end{array}$$

(10) $$\begin{array}{cc} \; & {\dot{Q}}_{\mathrm{sw}}={\dot{m}}_{\mathrm{sw}}c_{p,\mathrm{sw}}\left(T_{\mathrm{sw},\mathrm{out}}-T_{\mathrm{sw},\mathrm{in}}\right) \end{array}$$

(11) $$\begin{array}{cc} \; & {\dot{Q}}_{\mathrm{sw}}={\dot{Q}}_{\mathrm{con}} \end{array}$$

where $h_{1}$ is specific enthalpy of working fluid at condenser outlet, subscript con is condenser, and subscript sw is seawater.

The power consumption of the pump is calculated by

(12) $${\dot{W}}_{\mathrm{pu}}=\frac{{\dot{m}}_{r}\left(h_{2}-h_{1}\right)}{\eta_{\mathrm{pu}}}=\frac{{\dot{m}}_{r}\left(h_{2,\mathrm{is}}-h_{1}\right)}{\eta_{\mathrm{pu}}\eta_{\mathrm{pu},\mathrm{is}}}$$

where ${\dot{W}}_{\mathrm{pu}}$ is power consumption of the pump, $\eta_{\mathrm{pu}}$ is pump efficiency, and $\eta_{\mathrm{pu},\mathrm{is}}$ is isentropic efficiency of the pump.

The net power output of the SHEORC is calculated by

(13) $${\dot{W}}_{\mathrm{npo}}={\dot{W}}_{\exp}-{\dot{W}}_{\mathrm{pu}}$$

The net thermal efficiency of the SHEORC is obtained by

(14) $$\eta_{\mathrm{orc}}=\frac{{\dot{W}}_{\mathrm{npo}}}{{\dot{Q}}_{\mathrm{eg}}+{\dot{Q}}_{\mathrm{sa}}+{\dot{Q}}_{\mathrm{jw}}}$$

Exergy is the maximum production possible and indicates the energy value of the system. The exergy of each state point in the SHEORC can be obtained by

(15) $${\dot{E}}_{i}={\dot{m}}_{r}\left[\left(h_{i}-h_{0}\right)-T_{0}\left(s_{i}-s_{0}\right)\right]$$

where subscript *i* is each state point and $T_{0}$ is ambient temperature.

The exergy loss of each component in the SHEORC is expressed as [16]:

(16) $$\begin{array}{cc} \; & {\dot{I}}_{\mathrm{hea}}={\dot{E}}_{8}-{\dot{E}}_{7}+{\dot{E}}_{2}-{\dot{E}}_{3} \end{array}$$

(17) $$\begin{array}{cc} \; & {\dot{I}}_{\mathrm{heb}}={\dot{E}}_{10}-{\dot{E}}_{9}+{\dot{E}}_{3}-{\dot{E}}_{4} \end{array}$$

(18) $$\begin{array}{cc} \; & {\dot{I}}_{\mathrm{hec}}={\dot{E}}_{12}-{\dot{E}}_{11}+{\dot{E}}_{4}-{\dot{E}}_{5} \end{array}$$

(19) $$\begin{array}{cc} \; & {\dot{I}}_{\mathrm{con}}={\dot{E}}_{14}-{\dot{E}}_{13}+{\dot{E}}_{6}-{\dot{E}}_{2} \end{array}$$

(20) $$\begin{array}{cc} \; & {\dot{I}}_{\mathrm{ex}}={\dot{E}}_{5}-{\dot{E}}_{6}-{\dot{W}}_{\exp} \end{array}$$

(21) $$\begin{array}{cc} \; & {\dot{I}}_{\mathrm{pu}}={\dot{E}}_{1}-{\dot{E}}_{2}+{\dot{W}}_{\mathrm{pu}} \end{array}$$

where $\dot{I}$ is exergy loss of each component.

Based on the aforementioned calculation, the total exergy losses of SHEORC are calculated by

(22) $${\dot{I}}_{\mathrm{tot}}={\dot{I}}_{\mathrm{hea}}+{\dot{I}}_{\mathrm{heb}}+{\dot{I}}_{\mathrm{hec}}+{\dot{I}}_{\mathrm{con}}+{\dot{I}}_{\mathrm{ex}}+{\dot{I}}_{\mathrm{pu}}$$

then, the exergy efficiency of SHEORC can be expressed as [20]

(23) $${\dot{\eta }}_{\mathrm{ex}}=\frac{{\dot{W}}_{\mathrm{npo}}}{{\dot{I}}_{\mathrm{tot}}+{\dot{W}}_{\mathrm{npo}}}$$

### 2.5. Pinch Point Temperature Difference

**Figure 3:**
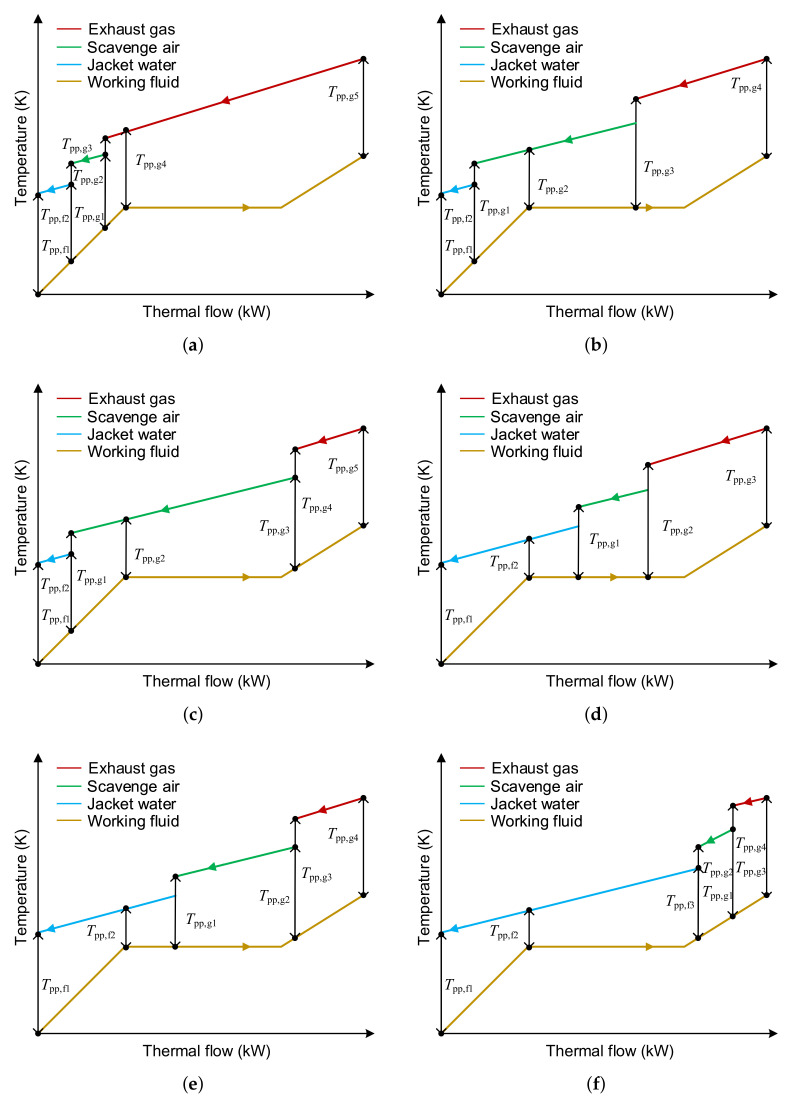
Probable situations in the calculation process of the PPTD when the SHEORC are utilizing three heat sources. (**a**) Preheated by JW and SA, evaporated and superheated by EG, (**b**) Preheated by JW and SA, evaporated by SA and EG, and superheated by EG, (**c**) Preheated by JW and SA, evaporated by SA, superheated by SA and EG, (**d**) Preheated by JW, evaporated by JW, SA, and EG, and superheated by EG, (**e**) Preheated by JW, evaporated by JW and SA, and superheated by SA and EG, (**f**) Preheated by JW, evaporated by JW, and superheated by JW, SA, and EG.

The temperature difference at the pinch point has a significant influence on heat exchanger performance and heat transfer. Therefore, pinch point temperature difference (PPTD) is a constraint when performing a thermodynamic analysis. In the calculation process, according to the amount of energy recovered in each heat exchanger, the calculation of the PPTD can be divided into 6 cases when the SHEORC are utilizing three waste heat sources. The six possible situations are shown in Figure 3. Note that when the heat source is jacket water, the type of the heat exchanger is a plate heat exchanger. If the heat source is SA or EG, shell and tube heat exchange was applied to recycle the waste heat source. Thus, as can be seen in Figure 3, the subscript f means that it is a plate heat exchanger, and the subscript g means shell and tube heat exchanger.

**Figure 4:**
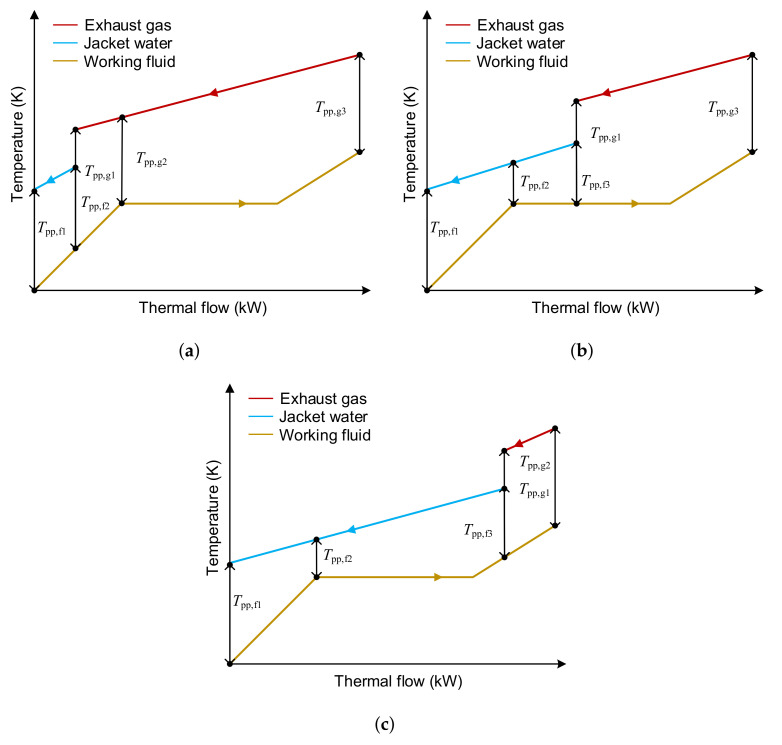
Probable situations in the calculation process of the PPTD when the SHEORC are utilizing two heat sources. (**a**) Preheated by JW and EG, evaporated and superheated by EG, (**b**) Peheated by JW, evaporated by JW and EG, superheated by EG, (**c**) Peheated by JW, evaporated by JW, superheated by JW and EG.

When the SHEORC is utilizing two waste heat sources, the calculation of the minimum PPTD could be divided into three circumstances. These conditions are shown in Figure 4. If the source combination is SA→EG, jacket water in Figure 4 should be replaced with scavenge air.

### 2.6. Heat Transfer Area

The Logarithmic Mean Temperature Difference (LMTD) method was applied to calculate heat transfer area. Thus, the heat transfer area of each heat exchanger, including condenser is calculated as:

(24) $$A_{i}=\frac{Q_{i}}{U_{i}\Delta T_{\mathrm{LM},i}F_{i}}$$

where, *F* is set as 0.95, $\Delta T_{\mathrm{LM}}$ is given as [16]

(25) $$\Delta T_{\mathrm{LM}}=\frac{\Delta T_{\max}-\Delta T_{\min}}{\ln(\Delta T_{\max}/\Delta T_{\min})}$$

Then, the $U_{\mathrm{pl}}$ of the plate heat exchanger is calculated by [16]

(26) $$\frac{1}{U_{\mathrm{pl}}}=\frac{1}{\alpha_{s}}+\frac{1}{\alpha_{r}}+\frac{\delta }{k}$$

The $U_{\mathrm{st}}$ of the shell and tube heat exchange is presented as [21]

(27) $$\frac{1}{U_{\mathrm{st}}}=\frac{1}{\alpha_{r,\mathrm{st},\mathrm{os}}}+\frac{d_{\mathrm{os}}}{\alpha_{s}d_{\mathrm{os}}}+\frac{d_{\mathrm{os}}\delta }{d_{\mathrm{ave}}k}+r$$

The heat transfer of the single phase working fluid in a plate heat exchange could be deduced by [22]

(28) $$\alpha_{c,\mathrm{pl}}=0.023\frac{k_{r}}{D_{e,\mathrm{pl}}}Re_{r}^{0.8}Pr_{r}^{0.4}{\left(\frac{\mu_{r}}{\mu_{w,r}}\right)}^{0.14}$$

The boiling heat transfer coefficient of working fluid in a counter flow vertical plate heat exchanger is expressed as [22]:

(29) $$\alpha_{\mathrm{tp},\mathrm{pl}}=1.926\frac{k_{r}}{D_{e,\mathrm{pl}}}Bo_{\mathrm{eq}}^{-0.3}Re_{\mathrm{eq}}^{0.5}Pr_{\mathrm{eq}}^{1/3}\left[\left(1-x\right)+x{\left(\frac{\rho_{l}}{\rho_{g}}\right)}^{0.5}\right]$$

The film condensation heat transfer coefficient of the working fluid in the plate exchanger is given as [23]

(30) $$\alpha_{\mathrm{con},\mathrm{pl}}=4.118\frac{k_{r,l}}{D_{e,\mathrm{pl}}}Re_{\mathrm{eq}}^{0.4}Pr_{l}^{1/3}$$

The heat transfer coefficient of the heat source in the plate heat exchange is calculated by [22]

(31) $$\alpha_{h,\mathrm{pl}}=0.2121\frac{k_{r}}{D_{e,\mathrm{pl}}}Re_{r}^{0.78}Pr_{r}^{1/3}{\left(\frac{\mu_{r}}{\mu_{w,r}}\right)}^{0.14}$$

The heat transfer coefficient of the single phase working fluid in shell and tube exchanger for 6000 < Re < $10^{7}$ and 0.5 < Pr < 120 is expressed as [24]:

(32) $$\alpha_{r,\mathrm{st}}=0.023\frac{k_{r}}{D_{e,\mathrm{st}}}Re_{r}^{0.8}Pr_{r}^{a}$$

where *a* is 0.4 for heating and *a* is 0.3 for cooling. The boiling heat transfer coefficient of working fluid in the tube is deducted as [25]:

(33) $$\alpha_{\mathrm{tp}}=\alpha_{l}\left[H_{1}H\;o^{H_{2}}{\left(25Fr_{\mathrm{lo}}\right)}^{H_{5}}+H_{3}Bo^{H_{4}}F_{\mathrm{fl}}\right]$$

where

(34) $$\begin{array}{cc} \; & \alpha_{l}=0.023\frac{k_{r}}{D_{e,\mathrm{st}}}{\left(\frac{G_{r}\left(1-x\right)D_{e,\mathrm{st}}}{\mu_{r}}\right)}^{0.8}{\left(\frac{c_{p,l}\mu_{r}}{k_{r}}\right)}^{0.4} \end{array}$$

(35) $$\begin{array}{cc} \; & H\;o={\left(\frac{1-x}{x}\right)}^{0.8}{\left(\frac{\rho_{g}}{\rho_{l}}\right)}^{0.5} \end{array}$$

$F\;r_{\mathrm{lo}}$ is Froude number, $F_{\mathrm{fl}}$ is fluid-dependent parameter, $H_{1}-H_{4}$ are depended on the value of Ho and given in Table 5.

The mean heat transfer coefficient for film condensation in horizontal tubes is expressed as [16]:

(36) $$\alpha_{\mathrm{con}}=0.943{\left[\frac{gk_{}^{3}\rho_{l}\left(\rho_{l}-\rho_{g}\right)\gamma }{D_{\mathrm{con}}\mu_{l}\left(T_{\mathrm{con}}-T_{w}\right)}\right]}^{1/4}$$

Finally, the heat transfer area of the heat exchangers can be deduced as

(37) $$\begin{array}{cc} A_{\mathrm{he},\mathrm{two}} & =A_{\mathrm{hea}}+A_{\mathrm{heb}} \end{array}$$

(38) $$\begin{array}{cc} A_{\mathrm{he},\mathrm{three}} & =A_{\mathrm{hea}}+A_{\mathrm{heb}}+A_{\mathrm{hec}} \end{array}$$

Since the heat transfer coefficient is different when the fluid is in different states. Therefore, the heat exchanger needs to be divided into several sections for calculating total heat transfer area.

### 2.7. Economic Model

In this article, we applied equipment module cost evaluation equations to evaluate the total cost of the presented SHEORC, including heat exchangers, expander, working fluid pump, and condenser. Therefore, the bare module cost $C_{\mathrm{BM}}$ of each component in the SHEORC is calculated as follows [26]:

(39) $$C_{\mathrm{BM},y}=C_{p,y}\left(B_{1,y}+B_{2,y}F_{M,y}F_{P,y}\right)$$

where, subscript *y* is equipment type including heat exchangers, expander, and pump, $C_{p}$ is purchased cost of the equipment at ambient pressure, $F_{P}$ is the pressure factor, $F_{M}$, $B_{1}$ and $B_{2}$ are empirical coefficients and shown in Table 6.

In Equation (39), $C_{p}$ for heat exchangers can be expressed as follows [26]

(40) $$\mathrm{lg}C_{p,y}=K_{1,y}+K_{2,y}\mathrm{lg}A_{n}+K_{3,y}{\left(\mathrm{lg}A_{y}\right)}^{2}$$

where, *A* is heat transfer area of the heat exchangers, $K_{1}$, $K_{2}$, and $K_{3}$ are empirical coefficients and are given in Table 6.

Furthermore, $F_{P}$ in Equation (39) is given by the following expression [26]:

(41) $$\mathrm{lg}F_{P,y}=C_{1,y}+C_{2,y}\mathrm{lg}P_{y}+C_{3,y}{\left(\mathrm{lg}P_{y}\right)}^{2}$$

where, *P* is the pressure in the equipment, $C_{1}$, $C_{2}$ and $C_{2}$ are empirical coefficients and are given in Table 6.

The purchased cost of equipment in the year of 2019 can be deduced from the cost of the year 2001 by using the the Chemical Engineering Plant Cost Index (CEPCI) and estimated as

(42) $$C_{\mathrm{BM},m,2019}=C_{\mathrm{BM},m,2001}\frac{{\mathrm{CEPCI}}_{2019}}{{\mathrm{CEPCI}}_{2001}}$$

where the value of ${\mathrm{CEPCI}}_{2019}$ is 607.5 [24], ${\mathrm{CEPCI}}_{2001}$ is 397.

Subsequently, the total capital expenditure is calculated by

(43) $$C_{\mathrm{tot}}=C_{\mathrm{BM},\mathrm{hea}}+C_{\mathrm{BM},\mathrm{heb}}+C_{\mathrm{BM},\mathrm{hec}}+C_{\mathrm{BM},\mathrm{con}}+C_{\mathrm{BM},\mathrm{ex}}+C_{\mathrm{BM},\mathrm{pu}}$$

Finally, levelized cost of energy (LCOE), which is an important metric of average cost of the electricity over lifetime, can be evaluated by [16]

(44) $$L\;C\;O\;E=\frac{C_{\mathrm{tot}}\cdot C\;R\;F+C\;O\;M}{t_{\mathrm{ot}}\cdot {\dot{W}}_{N\;P\;O}}$$

where

(45) $$C\;R\;F=\frac{i{\left(1+i\right)}^{LT}}{{\left(1+i\right)}^{LT}-1}$$

where LT is the life cycle time of SHEORC and is set to 20, the discount rate *i* is 4.9% [16], COM is the cost of operations and maintenance and is assumed as 1.5% of $C_{\mathrm{tot}}$, and $t_{\mathrm{ot}}$ is the operational time per year and is set as 8000 h [27].

## 3. Optimization Process

### 3.1. Optimization Algorithms

In the present paper, the Genetic Algorithm (GA) [28] method was employed for the single objective optimization process [29]. GA is generally applied to find the optimal solutions to optimization by mutation, crossover and selection. The Non-dominated Sorting Genetic Algorithm II (NSGA II) [30], which has high computational efficiency, was applied to solve the multi-objective optimization problem by providing a Pareto Frontier set. The Technique for Order Preference by Similarity to Ideal Solution (TOPSIS) was used to find the optimal solution on the Pareto Frontier. The TOPSIS process is given as follows:

Step 1: Find the maximum values $X^{+}$ and minimum values $X^{-}$ on the Pareto Frontier, this step could be described as:

(46) $$\begin{array}{cc} X^{+} & =(\max\left\{x_{11},x_{21},\ldots ,x_{n1}\right\},\max\left\{x_{12},x_{22},\ldots ,x_{n2}\right\},\ldots ,\max\left\{x_{1m},x_{2m},\ldots ,x_{nm}\right\}) \end{array}$$

(47) $$\begin{array}{cc} \; & =(X_{1}^{+},X_{2}^{+},\ldots ,X_{m}^{+}) \end{array}$$

(48) $$\begin{array}{cc} X^{-} & =(\min\left\{x_{11},x_{21},\ldots ,x_{n1}\right\},\min\left\{x_{12},x_{22},\ldots ,x_{n2}\right\},\ldots ,\min\left\{x_{1m},x_{2m},\ldots ,x_{nm}\right\}) \end{array}$$

(49) $$\begin{array}{cc} \; & =(X_{1}^{-},X_{2}^{-},\cdots ,X_{m}^{-}) \end{array}$$

where, *x* is the objective values for each individual on the Pareto Frontiers, subsript *n* is the number of individuals, and subscript *m* is the number of objectives.

Step 2: Compute the Euclidean distances *D* of each solution between the ideal solution:

(50) $$\begin{array}{c} D_{i}^{+}=\sqrt{\sum_{j=1}^{m}{\left(X_{j}^{+}-x_{ij}\right)}^{2}} \end{array}$$

(51) $$\begin{array}{c} D_{i}^{-}=\sqrt{\sum_{j=1}^{m}{\left(X_{j}^{-}-x_{ij}\right)}^{2}} \end{array}$$

Step 3: Calculate the relative closeness $\xi_{i}$ of each solution. This step can be presented as:

(52) $$\xi_{i}=\frac{D_{i}^{-}}{D_{i}^{+}+D_{i}^{-}}$$

Step 4: Selecting the solution which has the highest value $\xi_{i}$ as the optimal solution.

The parameters of GA and NSGA II are shown in Table 7.

### 3.2. Objective Functions and Decision Variables

Thermodynamic and economic performance are the two most significant objectives in designing a waste heat recovery system. Thus, in the single-objective optimization, power output was selected as the objective. In the multi-objective optimization, Equations (44) and (23) were set as the objectives.

Meanwhile, this article picked parameters that included the outlet temperature of the waste heat source $T_{\mathrm{eg},\mathrm{out}},T_{\mathrm{sa},\mathrm{out}},T_{\mathrm{jw},\mathrm{out}}$, super-heat temperature $T_{\sup}$, evaporating temperature $T_{\mathrm{ev}}$, condensation temperature $T_{\mathrm{con}}$, and pinch point temperature in the condenser $T_{\mathrm{pp},\mathrm{con}}$ as decision variables.

### 3.3. Constraints

Constraints in the optimization are general constraints and boundaries on variables. The exhaust gas temperature should not below the dew point temperature $T_{\mathrm{dew}}$. The $T_{\mathrm{dew}}$ could be calculated as follows [14]:

(53) $$T_{\mathrm{dew}}=203.25+27.6\mathrm{lg}\left(P_{H_{2}O}\right)+10.83\mathrm{lg}\left(P_{{\mathrm{SO}}_{3}}\right)+1.06{\left(\mathrm{lg}(P_{{\mathrm{SO}}_{3}})+8\right)}^{2.19}$$

where $P_{{\mathrm{SO}}_{3}}$ and $P_{H_{2}O}$ are partial pressures of ${\mathrm{SO}}_{3}$ and $H_{2}O$, respectively.

The other constraints, boundaries and parameters of the SHEORC model are given in Table 8.

## 4. Results and Discussion

### 4.1. Model Validation

The thermodynamic and economic model of SHEORC were implemented in MATLAB 2016a with the CoolProp 6.41 [33]. The thermodynamic properties of the waste heat sources and working fluids are provided by the software CoolProp. The model realized in MATLAB was validated with the results presented in the Ref. [20]. The comparison results are listed in Table 9. The difference may be due to the calculation method of the heat transfer coefficient and the thermodynamic properties.

### 4.2. Effects of the Mass Flow Rate and Evaporating Pressure on SHEORC Performance

The mass flow rate of the working fluid and the evaporating pressure significantly influence the thermodynamic and economic performance. Therefore, in this section, the influence of the combination of these two variables was investigated. The proportion of energy recovered in each heat exchanger is based on the amount of heat that each source carried and is set as fixed in the analysis.

**Figure 5:**
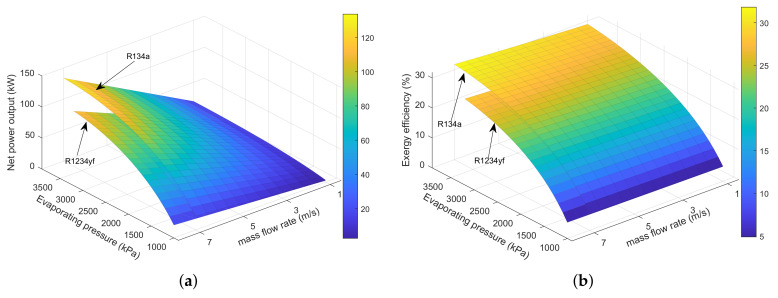

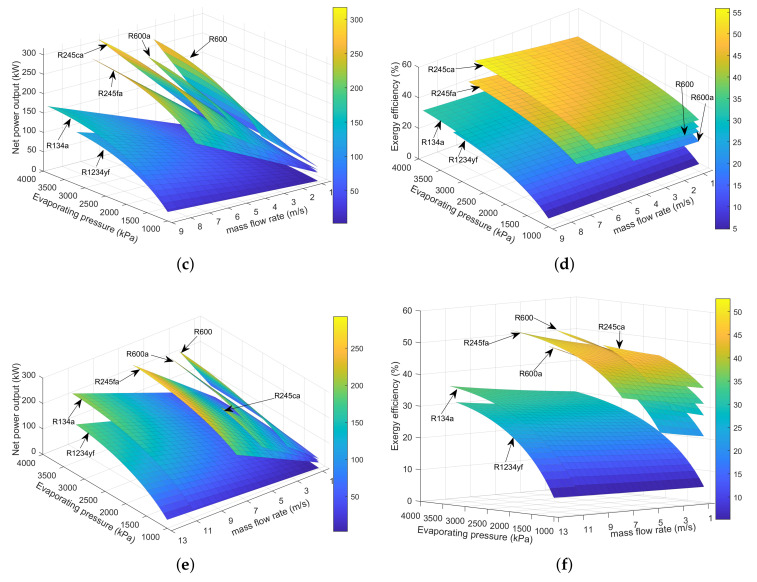
Effects of mass flow rate and evaporating temperature on (**a**) JW→EG power output, (**b**) JW→EG exergy efficiency, (**c**) SA→EG power output, (**d**) SA→EG exergy efficiency, (**e**) JW→SA→EG power output, (**f**) JW→SA→EG exergy efficiency.

Figure 5 illustrates the influence of mass flow rate and evaporating pressure on power output and exergy efficiency of the SHEORC using different working fluids. As can be seen, the mass flow rate and power output have a positive linear relationship under the same evaporating pressure. These results, caused by increasing the mass flow rate, increases the amount of energy recovered in each heat exchanger. The mass flow rate does not affect the exergy efficiency of the cycle. The results indicated that the exergy efficiency of the ORC is only related to the parameters of the cycle itself. The increase in evaporating pressure causes an increase in the power output and exergy efficiency. The increasing rate decreases with the increase of evaporation pressure. These results suggested that an increase in the mass flow rate and evaporating pressure could increase power output and exergy efficiency.

Additionally, it is viewed that in the JW→EG condition, R134a has a better thermodynamic performance than R1234yf. Furthermore, R245fa, R245ca, R600, and R600a are not suitable for the JW→EG condition. These results were due to the high evaporating temperature of these working fluids. In the SA→EG situation, it is observed that R600 has the best performance under the same mass flow rate and evaporating pressure. However, the R245ca has the maximum power output and exergy efficiency. The reason was that the critical temperature of the working fluid limits the power output. In the JW→SA→EG circumstance, it can be seen that R245fa has the highest power output and exergy efficiency. This result was caused by the fact that the addition of jacket water limits the increase in the evaporation temperature of R245ca. The results matched with the previous results in article [7]. The analysis results suggested that the suitable working fluid for JW→EG, SA→EG, and JW→SA→EG is R134a, R245ca, and R245fa, respectively.

**Figure 6:**
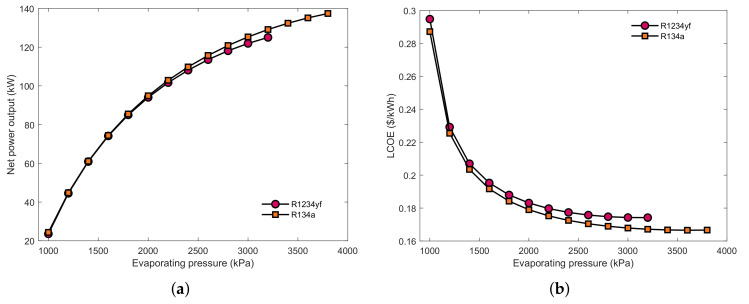

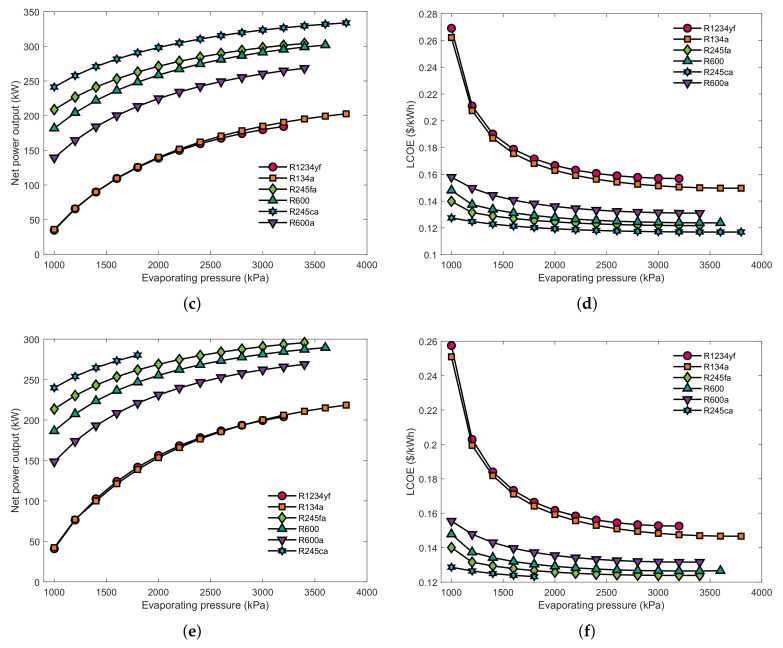
Effects of the evaporating temperature on (**a**) JW→EG power output, (**b**) JW→EG LCOE, (**c**) SA→EG power output, (**d**) SA→EG LCOE, (**e**) JW→SA→EG power output, (**f**) JW→SA→EG LCOE under the optimal mass flow rate.

Figure 6 presents that the influence of the evaporating pressure on the power output and LCOE under the optimal mass flow rate. As can be seen, increasing the evaporating pressure leads to an increase in power output and a decrease in LCOE. In the JW→EG condition, R134a had a better thermoeconomic performance than R1234yf. In the SA→EG situation, R245ca performed the best both in thermodynamic and economic indicators. However, in the JW→SA→EG combination, R245fa had the highest power output and lowest LCOE. These results are consistent with the results shown in Figure 5. Additionally, it can be seen that combining the jacket water into the waste heat source group causes a deterioration in the performance of the SHEORC. Furthermore, it is viewed that due to the addition of jacket water, R245ca could not arrive at high evaporation pressure. That is why R245fa has the best performance in the JW→SA→EG circumstance.

### 4.3. Effects of the Waste Heat Recovery Proportion on SHEORC Performance

According to the results reported in Section 4.2, adding the jacket water resulted in a worse performance of the SHEORC. Therefore, in this section, the effect of heat recovery proportion on the performance of the SEHORC was investigated.

**Figure 7:**
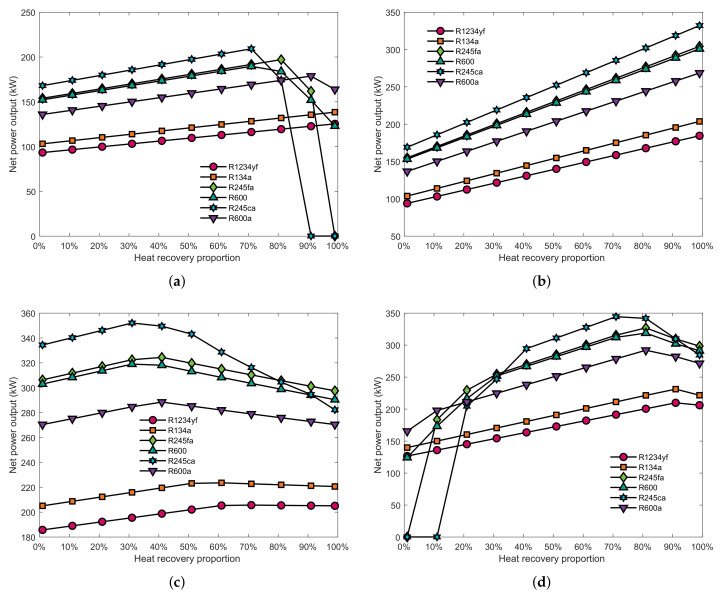
Effect of the heat recovery proportion on power output (**a**) Heat recovery proportion of the jacket water in JW→EG, (**b**) Heat recovery proportion of scavenge air in SA→EG, (**c**) Heat recovery proportion of jacket water in JW→SA→EG, (**d**) Heat recovery proportion of scavenge air in JW→SA→EG.

Figure 7 shows the effect of the heat recovery proportion on the power output of the SHEORC. As can be seen in Figure 7a, when the SHEORC are using R1234yf or R134a as working fluid, increasing the energy proportion of the jacket water causes an increase of the power output. However, when using other working fluids, the power output will rise initially, and then it will drop. The power output of the SHEORC using R245fa or 245ca drops to zero due to the PPTD limit. This result indicated that there is an optimal heat recovery proportion of the jacket water in the JW→EG condition. Figure 7b illustrates that the increase in heat recovery proportion of scavenge air would increase power output in the SA→EG combination. This result revealed that these two sources have good compatibility.

The result indicated that adding jacket water into the SA→EG combination could increase the power output of the SHEORC. It can be seen in Figure 7c, as the energy proportion of jacket water increases, the power output rises at the beginning and then begins to fall. It is viewed that integrating scavenge air with JW→EG groups could change the pinch point position and improve the power output. However, as can be seen, there is also an optimal heat recovery proportion of scavenge air. These results suggested that the heat recovery proportion of the waste heat sources should be optimized to get the best performance.

### 4.4. Single Objective Optimization

In this section, a single objective optimization was conducted to find the maximum power output of the SHEORC under each heat source combination. The optimization results are shown in Table 10.

As can be seen from Table 10, in the JW→EX, the SHEORC using R245ca has the maximum power output. Interestingly, when SHEORC uses R245fa as the working fluid, the outlet temperature of the jacket water is lower than when SHEORC is using R245ca as the working fluid. In other words, the SHEORC using R245fa recovered more energy from the waste heat sources, but the power output is lower than the SHEORC using R245ca. This result could also be deduced from the thermal efficiency of the SHEORC. When the SHEORC is applied to utilizing the SA→EX, the SHEORC using R245ca has the highest power output. In the JW→SA→EX circumstance, the SHEORC using R245ca has the highest power output. However, the power output of the SHEORC using R245ca or R245ca increases little when the SHEORC was integrating the jacket water into the system.

### 4.5. Multi-Objective Optimization

In this section, bi-objective optimization based Pareto frontier solution is applied to maximize $\eta_{\mathrm{ex}}$ and minimize LCOE simultaneously. The Pareto Frontiers for all the working fluids are shown in Appendix A. The parameters of the SHEORC with the optimal solution provided by the TOPSIS method are listed in Table 11.

**Figure 8:**
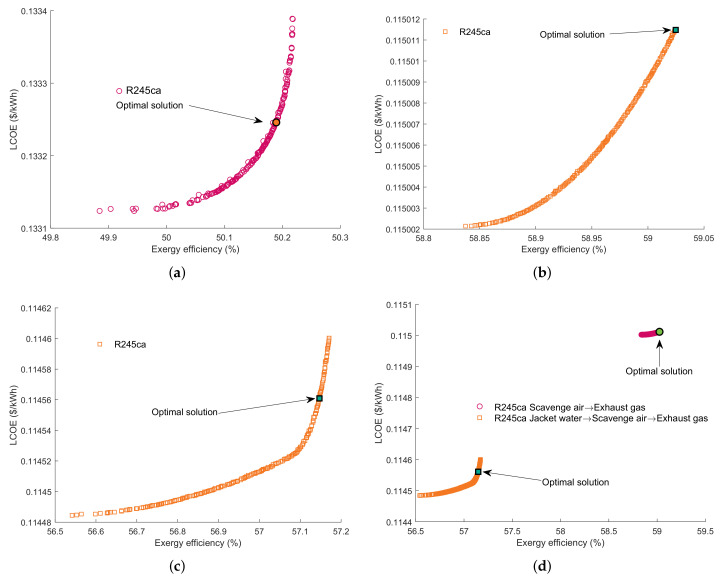
Pareto frontiers for each source combination. (**a**) JW→EG, (**b**) SA→EG, (**c**) JW→SA→EG, (**d**) Comparison between JW→SA→EG and SA→EG.

Figure 8 presents the Pareto Frontiers set and optimal solutions of the most suitable working fluid in each source combination. As can be seen, it is impossible to achieve a maximum $\eta_{\mathrm{ex}}$ and minimum LCOE simultaneously. The SHEORC using R245ca as the working fluid has the best performance in all source combinations. The results indicated that in the SHROC, the suitable working fluid might be decided by the temperature of the last heat source. As can be seen from Table 11, in the SA→EX source combination, the SHEORC has a higher exergy efficiency and power output. The SHEORC utilizing the JW→SA→EX has a lower LCOE. However, the SHEORC with SA→EX has the highest power output. The optimization results suggested that it may be better to recover SA→EX by using SHEORC to recycle the waste heat sources of the marine two-stroke diesel engines.

## 5. Conclusions

This study has proposed a novel Series Heat Exchangers Organic Rankine Cycle (SHEORC) to recover energy from three waste heat sources of the marine diesel engine. The effects of the working fluid, evaporating pressure, and heat recovery proportion on the thermoeconomic performance of the SHEORC with three source combinations are investigated. The single objective and bi-objective optimizations were conducted to find the optimal parameters for working fluid of the SHEORC. Based on the analysis and optimizations, the conclusions can be drawn as follows:

1. With the increase of the working fluid’s mass flow rate, the power output of the SHEORC with three various source combinations will increase. However, increasing the mass flow rate of the working fluid does not affect the exergy efficiency of the SHEORC. The evaporating pressure has a positive effect on both thermodynamic and economic performance of the SHEORC;
2. With JW→EG and JW→SA→EG groups, there are optimal heat recovery proportions under which the SHEORC could obtain the best performance;
3. In the single-objective optimization, R245ca was the suitable working fluid for all source combinations. The power output of SA→EG and JW→SA→EG groups are similar. Integrating the jacket water into the SA→EG group would not increase the power output.
4. In the bi-objective optimization, JW→SA→EG has a little lower LCOE, and SA→EG has higher power output and exergy efficiency. Thus, using SHEORC to recover energy from scavenge air and exhaust gas may be the best choice for marine waste heat recovery.

This paper provides guidelines for the marine waste heat recovery system using Series Heat Exchangers Organic Rankine Cycle. It is viewed that the SHEORC may be unable to recover jacket water, scanvege air, and exhaust gas simultaneously. Therefore, further work will be focused on using multi-loop ORC to recycle the waste heat sources from the marine two-stroke diesel engine.

## Figures and Tables

**Table 1:** Basic parameters of the diesel engine.

Parameter	Value
Type	6S35ME-B9
Bore (mm)	350
Stroke (mm)	1550
Cylinders	6
Compression ratio	21
Firing order	1-5-3-4-2-6
Brake power (kW)	3570
Engine speed (rpm)	142

**Table 2:** The mass flow and temperature of the heat resources at Specified Maximum Continuous Rating (SMCR).

Parameter	Unit	Value
Exhaust gas temperature (after turbine)	K	535.75
Scavenge air temperature (after compressor)	K	422.37
Jacket water temperature	K	353.15
Exhaust gas mass flow (after turbine)	kg/s	10.33
Scavenge air mass flow (after compressor)	kg/s	10.45
Jacket water mass flow	kg/s	11.86

**Table 3:** Composition of exhaust gas (%mol).

Parameter	Value
$O_{2}$	13.75
$N_{2}$	75.812
${\mathrm{CO}}_{2}$	4.703
$H_{2}O$	4.771
Ar	0.902
${\mathrm{SO}}_{2}$	0.062

**Table 4:** The properties of the working fluids.

Item	R1234yf [15]	R134a [16]	R600 [17]	R600a [18]	R245ca [18]	R245fa [19]
Molar mass (kg/kmol)	114.04	102.03	58.12	58.12	134.05	134.05
Boiling temperature (K)	243.7	247.1	272.7	261.4	298.4	288.3
Critical temperature (K)	367.85	374.21	425.125	407.8	447.57	427.16
Critical pressure (kPa)	3382	4059	3796	3629	3941	3651
ODP	0	0	0	0	0	0
GWP	4	1430	20	20	610	1030
ASHRAE 34 safety group ^a^	A2	A1	A3	A3	A1	B1

^a^ 1: No flame propagation; 2: Lower flammability; 3: Higher flammability; A: Lower toxicity; B: Higher toxicity.

**Table 5:** Constants in the Kandlikar correlation [25].

Constant	Convective Region	Nucleate Boiling Region
$H_{1}$	1.1360	0.6683
$H_{2}$	−0.9	−0.2
$H_{3}$	667.2	1058.0
$H_{4}$	0.7	0.7
$H_{5}$ *	0.3	0.3

* $H_{5}=0$ for vertical tubes, and for horizontal tubes with $F\;r_{\mathrm{lo}}>0.04$.

**Table 6:** Coefficients in module cost evaluation equations [26].

Equipment Type	$K_{1}$	$K_{2}$	$K_{3}$	$C_{1}$	$C_{2}$	$C_{3}$	$B_{1}$	$B_{2}$	$F_{M}$
Plate heat exchanger	4.6561	−0.2947	0.2207	0	0	0	0.96	1.21	1
Shell and tube heat exchanger	4.3247	−0.3030	0.1634	0.0381	−0.11272	0.08183	1.63	1.66	1.2
Condenser	4.6561	−0.2947	0.2207	0	0	0	0.96	1.21	1
Expander	2.2476	1.4965	−0.1618	0	0	0	/	/	3.8
Working pump	3.3892	0.0536	0.1538	−0.3935	0.3957	−0.00226	1.89	1.35	1.6

**Table 7:** Critical parameters of the GA and NSGA II method.

Items	GA	NSGA II
Population size	50	100
Maximum iterations	100	200
Crossover probability	0.75	0.75 [31]
Mutation probability	0.25	0.25 [31]

**Table 8:** Main parameters of the thermodynamic model and decision boundaries.

Items	Value	Unit
Pump isentropic efficiency [32]	75	%
Expander isentropic efficiency [17]	80	%
Environment temperature [16]	20	℃
Exhaust gas outlet temperature	≥$T_{\mathrm{dew}}$	℃
Jacket water outlet temperature	≥70	℃
Scavenge air outlet temperature	≥45	℃
Seawater temperature	20	℃

**Table 9:** Comparison results of the economic model with the previous article [20].

Items	$T_{\mathrm{ev}}$ (K)	$T_{\mathrm{con}}$ (K)	$T_{\sup}$ (K)	$T_{\mathrm{pp},\mathrm{ev}}$ (K)	$T_{\mathrm{pp},\mathrm{con}}$ (K)	LCOE ($/kWh)	$\eta_{\mathrm{exer}}$ (%)
State B [20]	372.00	303.01	8.32	4.39	5	0.103	52.71
R134a (Present)	372.00	303.01	8.32	4.39	5	0.110	52.81
State D [20]	394.45	303.40	4.46	3	5	0.131	56.44
R11 (Present)	394.45	303.40	4.46	3	5	0.135	56.51

**Table 10:** Single objective optimization results.

Source Group	$W_{\mathrm{npo}}$/kW	$\eta_{\mathrm{orc}}$/%	Fluid	$T_{\mathrm{ev}}$/K	$T_{\sup}$/K	$T_{\mathrm{con}}$/K	$T_{\mathrm{jw},\mathrm{out}}$/K	$T_{\mathrm{sa},\mathrm{out}}$/K	$T_{\mathrm{eg},\mathrm{out}}$/K
JW→EX	200.65	16.30	R245ca	432.68	20.97	305.15	348.58	-	447.95
JW→EX	193.66	15.34	R245fa	421.18	15.95	305.15	347.94	-	447.95
SA→EX	354.19	16.76	R245ca	444.57	8.78	305.15	-	318.15	447.95
SA→EX	329.78	15.60	R245fa	424.16	32.33	305.15	-	318.15	447.95
JW→SA→EX	355.18	16.75	R245ca	444.57	8.40	305.15	351.55	324.98	447.95
JW→SA→EX	330.58	15.58	R245fa	424.16	26.36	305.15	352.27	321.51	447.95

**Table 11:** The parameters of the SHEORC with the optimal solution on the Pareto frontier set.

Source Combination	JW→EX	SA→EX	JW→SA→EX
$W_{\mathrm{npo}}$/kW	207.17	354.19	350.98
$\eta_{\mathrm{orc}}$/%	16.86	16.76	16.76
$\eta_{\mathrm{ex}}$/%	50.18	59.02	57.15
LCOE	0.1332	0.1150	0.1146
$T_{\mathrm{ev}}$/K	444.57	444.57	444.57
$T_{\sup}$/K	16.48	8.78	8.95
$T_{\mathrm{con}}$/K	305.15	305.15	305.15
$T_{\mathrm{pp},\mathrm{con}}$/K	11	11	11
$T_{\mathrm{jw},\mathrm{out}}$/K	348.59	-	345.25
$T_{\mathrm{sa},\mathrm{out}}$/K	-	318.15	356.84
$T_{\mathrm{eg},\mathrm{out}}$/K	447.95	447.95	447.95

**Figure A1:**
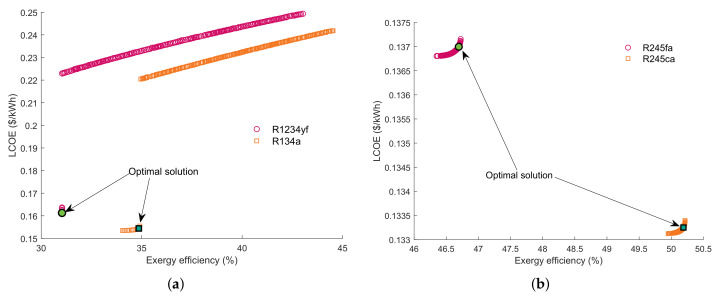

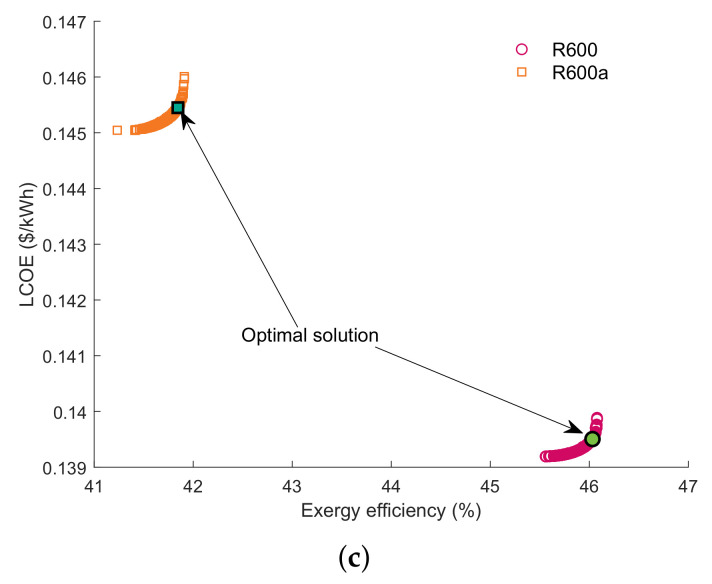
Pareto Frontiers for the working fluids in JW→EG condition. (**a**) R1234yf and R134a, (**b**) R245fa and R245ca, (**c**) R600 and R600a.

**Figure A2:**
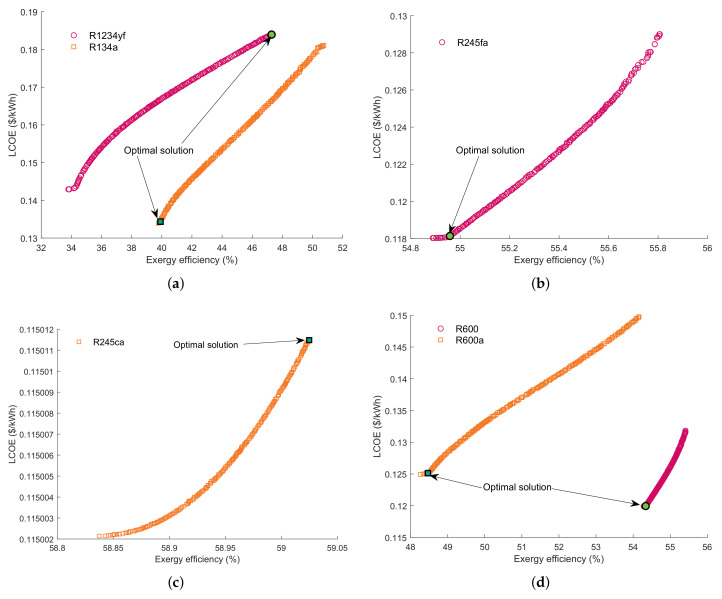
Pareto frontiers for the working fluids in SA→EG condition. (**a**) R1234yf and R134a, (**b**) R245fa, (**c**) R245ca, (**d**) R600 and R600a.

**Figure A3:**
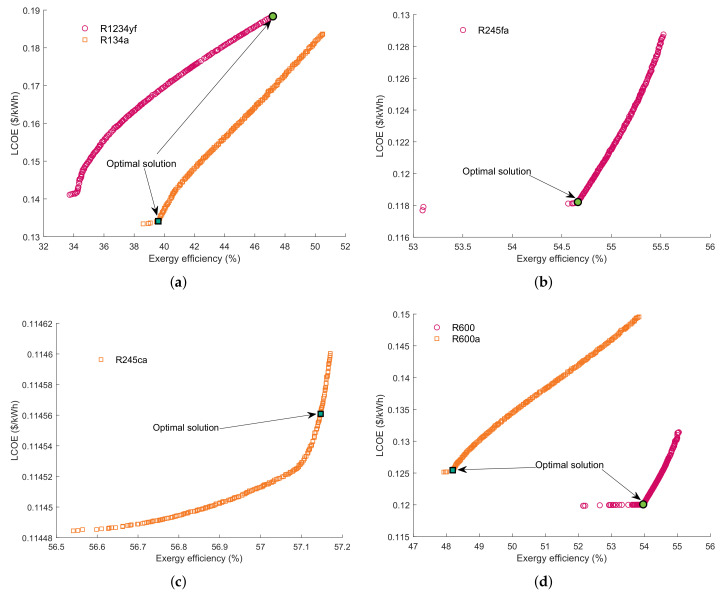
Pareto frontiers for the working fluids in JW→SA→EG condition. (**a**) R1234yf and R134a, (**b**) R245fa, (**c**) R245ca, (**d**) R600 and R600a.

## Data Availability

Data is contained within the article.

## Abbreviations

The following abbreviations are used in this manuscript:

ORC	Organic Rankine Cycle
SHEORC	Series Heat exchanges Organic Rankine Cycle
LCOE	levelized cost of energy
HFO	Heavy Fuel Oil
DLORC	Dual-Loop Organic Rankine Cycle
JW	Jacket water
EG	Exhaust gas
SA	Scavenge air
SMCR	Specified Maximum Continuous Rating
PPTD	Pinch point temperature difference
LMTD	Logarithmic Mean Temperature Difference
CRF	Capital recovery factor
NSGA II	Non-dominated Sorting Genetic Algorithm II
GA	Genetic Algorithm
TOPSIS	Technique for Order Preference by Similarity to Ideal Solution
CEPCI	Chemical Engineering Plant Cost Index
GWP	Global Warming Potential
ODP	Ozone Depletion Potential
COM	Cost of operation

## Symbols

The following symbols are used in this manuscript:

*Q*	Heat transfer rate, kW
*T*	Temperature, K
$\dot{m}$	Mass flow rate, kg/s
$c_{p}$	Specific heat, kJ/(kg K)
*W*	Power, kW
$\dot{E}$	Exergy, kW
$\dot{I}$	Exergy destruction rate, kW
*h*	Specific enthalpy, kJ/kg
*i*	Interest rate
Re	Reynolds number
Pr	Prandtl number
Fr	Froude number
$D_{e}$	Equivalent diameter, m
Bo	Boling number
$C_{\mathrm{BM}}$	Bare module equipment cost
$C_{p}$	Bare module cost for ambient pressure and carbon steel construction
$F_{P}$	Pressure factor
*K*	Contraction and expansion loss coefficients
*U*	Total heat transfer coefficient, W/m${}^{2}$ K
*A*	Heat transfer area, $m^{2}$
LT	Life time
*t*	time, h
*k*	Thermal conductivity, W/m${}^{2}$ K
*g*	Acceleration of gravity, $m/s^{2}$
δ	Thickness of tube or plate, m
η	Efficiency, %
α	Heat transfer coefficient, W/m${}^{2}$ K
μ	Viscosity, Pa·s
ρ	Density, kg/m${}^{3}$
ξ	Relative closeness
γ	Latent heat, J/kg

## Subscripts

The following subscripts are used in this manuscript:

s	source
ex	Exergy
in	Inlet
tot	total
w	Wall
out	Outlet
ev	Evaporator
sup	Superheat
exp	Expander
is	Isentropic
con	Condenser
sw	Seawater
pp	Pinch point
dew	Dew point
pl	Plate heat exchanger
npo	Net power output
st	Shell and tube heat exchanger
max	Maximum
min	Minimum
r	working fluid
w	Wall
l	Liquid
g	Gas
eq	Equivalent
os	Outside
hea	Heat exchanger A
heb	Heat exchanger B
hec	Heat exchanger C
